# 

**Published:** 1904-04

**Authors:** 


					﻿SUBSCRIPTION 9Trt
PUBLISHED MONTgV?
ATLANTA
JOURNAL-RECORD
Wn'^'OF MEDICINE. _
Vol. VI.
Atlanta, Ga., ApriI, 1904.

				

